# The single most important lesson from COVID-19 – It is time to take public health seriously

**DOI:** 10.7189/jogh.11.03073

**Published:** 2021-04-24

**Authors:** Sathyanarayanan Doraiswamy, Sohaila Cheema, Amit Abraham, Marco Ameduri, Ravinder Mamtani

**Affiliations:** 1 Institute for Population Health, Weill Cornell Medicine – Qatar, Doha, Qatar; 2 Weill Cornell Medicine – Qatar, Doha, Qatar

Public health is “the art and science of preventing disease, prolonging life and promoting health through the organized efforts of society” [1]. In the 20th century, progressive decline in death rate and improvement in life expectancy (25 out of 30 years) have been attributed predominantly to advances in public health.

In contrast to public health, medical care focuses on therapeutic solutions. Finding the optimum balance between the medical and public health approaches in complex situations is necessary and yet challenging. The absence of such a balance may lead to misplaced and misdirected health policies and priorities that can be detrimental to population health. The COVID-19 pandemic has tested the capability of public health systems worldwide to protect populations. It has highlighted a glaring lack of global consensus on how to implement basic health measures to deal with the pandemic – a notable example being lives lost in nursing care home populations, which should have been prioritized at the onset, but were not [2]. It has also brought to light the need for effective and meaningful communication of emerging scientific evidence for populations to better understand the pandemic, its risks, and how to better minimize them.

In this viewpoint, we evaluate the commitment to public health in the last decade, focusing on three domains: public health in medical education, investments in public health and prevention programs, and public health research.

## PUBLIC HEALTH IN MEDICAL EDUCATION

We explored the number of hours medical students spend learning public health in medical curricula. We could not find any meaningful literature discussing ‘public health’ in this context as such. We then examined medical school curricula for topics that underpin public health, such as preventive medicine, environmental health, lifestyle health, health policy and systems. Several studies reported that many medical schools offer limited training in occupational and environmental health [3]. Lack of awareness about environmental and occupational risks compromises physicians’ ability to effectively manage their patients and adequately protect themselves from occupational risks they face. The impact of COVID-19 on the health care workforce is well documented [4]. For instance, the mortality among physicians particularly among the Black, Asian, and Minority physicians is of serious concern. Unaddressed metabolic risk factors, inadequate training in infection prevention, and limited access to personal protective equipment (PPE) were cited as reasons for this higher mortality [5].

**Figure:**
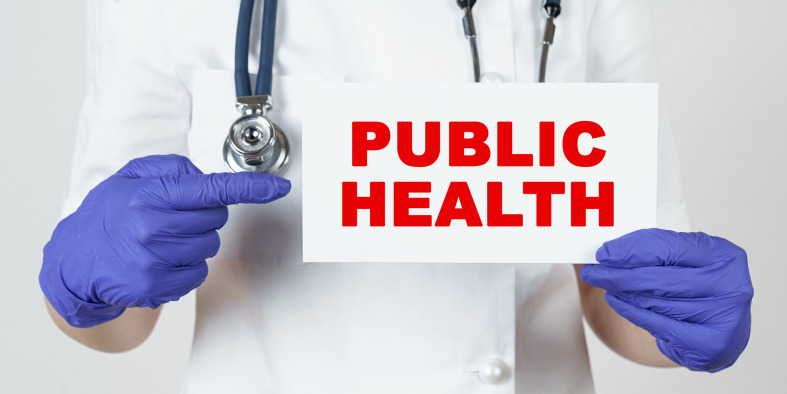
Photo: By SkazovD, via Shutterstock.

Patel et al. (2014) found that 40% of students in the United States of America (US) reported inadequate instruction in health policy, and that only a minimal improvement had been observed over the years [6]. Physicians need leadership and advocacy skills to support the health care needs of the populations they serve. Medical education programs are inadequate in providing such skills, critical to advancing and promoting public health [7]. A United Kingdom study established that while students were interested in receiving leadership and management education, the existing curriculum was deficient in this area [8]. These inadequacies have had repercussions during the COVID-19 pandemic. Physicians faced difficulty advocating for their own PPE access, while others in managerial positions were oblivious to their peer needs, demonstrating a lack of leadership and empathy — key skills imparted by public health education.

Satisfactory medical education would require a robust public health curriculum that is practical and field-oriented, well-qualified teachers and facilitators, and collaborators who provide students with real-world experience. Currently, such a model is elusive, with heterogeneity and fragmentation presenting a challenge to teaching and evaluating public health education in medical schools.

## INVESTMENT IN PUBLIC HEALTH AND PREVENTION PROGRAMS

Globally, improvements in national health budget allocation and investment in public health has translated into an increase in life expectancy and a reduction in maternal and infant mortality albeit with a slow minimalistic approach. In 2016, public health (preventive care) accounted for just 12% of global health expenditure [9], while in 2017 the Organization for Economic Cooperation and Development (OECD) countries invested only 2.8% of their total health expenditure in public health [10]. In 2018, only 3% of all US health care spending was allocated to public health. **Table 1** quantifies preventive care (as a surrogate of public health) investment as a percentage of GDP and total health expenditure made by OECD countries. We ranked countries (high to low) according to the share of their preventive care investment within the overall health portfolio for 2018 and then included the top five countries — data available for OECD (2010-2018).

**Table 1:** Investments in preventive care in OECD countries

Investment in preventive care (% of GDP)												
**Country**	**2010**	**2011**	**2012**	**2013**	**2014**	**2015**	**2016**	**2017**	**2018**	**Absolute change**	**Mean**	**SD**
Canada	0.63	0.61	0.61	0.60	0.61	0.62	0.64	0.63	0.64	0.02	0.62	0.01
Italy	0.26	0.24	0.36	0.37	0.38	0.37	0.37	0.38	0.38	0.12	0.34	0.05
Finland	0.30	0.29	0.31	0.32	0.32	0.38	0.37	0.36	0.36	0.07	0.33	0.03
Korea	0.20	0.20	0.22	0.23	0.23	0.26	0.25	0.28	0.26	0.06	0.24	0.03
Estonia	0.22	0.19	0.20	0.18	0.19	0.20	0.21	0.21	0.22	0.00	0.20	0.01
**Investment in preventive care (% of current health spending)**												
**Country**	**2010**	**2011**	**2012**	**2013**	**2014**	**2015**	**2016**	**2017**	**2018**	**Absolute change**	**Mean**	SD*
Canada	5.87	5.90	5.81	5.83	5.91	5.83	5.81	5.81	5.96	0.09	5.86	0.05
Italy	2.86	2.76	4.10	4.16	4.27	4.17	4.22	4.41	4.41	1.55	3.93	0.64
Finland	3.23	3.17	3.23	3.23	3.27	3.88	3.98	3.96	3.98	0.75	3.55	0.38
Korea	3.36	3.35	3.55	3.62	3.60	3.96	3.67	3.90	3.48	0.12	3.61	0.21
Estonia	3.49	3.34	3.53	3.01	3.06	3.18	3.22	3.12	3.30	-0.19	3.25	0.18

SD – standard deviation

Among the OECD countries, Canada had the highest investment, spending 6% of its total health spending in 2018 on public health interventions/programs [11]. The non-OECD countries seemingly spend a larger proportion of their total health spending on preventive care, but this often is the context of a smaller GDP and, consequently, smaller overall allocated health budget [12]. The trends demonstrate either plateauing or stagnating investment in preventive care, in spite of the staggering returns of public health interventions in high income nations, currently at 14.3:1 [13] – and likely similar in low and middle income nations. Enhanced governmental spending in the public health sector can stimulate long-term economic growth, and improve health, which will consequently result in lower overall health care costs. At an individual level, the link between health investment on the one hand and productivity and income on the other are indubitable.

## PUBLIC HEALTH RESEARCH

The National Institute of Health (2012-2017) research portfolio analysis found that only 16.7% of projects and 22.6% of the total research allocation were for primary and secondary prevention research in the US, with less than 5% of the projects choosing an outcome related to the leading risk factors for death and disability in the country. The funded projects were mostly observational and secondary research and only a few intervention-focused [14]. Inadequate investment in public health research presents a challenge in high-income countries and has serious consequences in low and low-middle income countries as more targeted high-impact investments are needed, given the resource limitations.

Public health research should be a part of a systematic process in which available resources are directed to finding solutions to major health challenges confronting a country. In general, countries lack a transparent prioritization process that ensures the available meagre funding is invested in high impact areas [15]. The complexity of issues surrounding public health propels researchers to focus their efforts on easy-to-conduct research – for example cross-sectional studies – with limited utility. In our opinion, such studies, particularly with repetitive or inconclusive findings are costly and may take away the focus from more significant and useful studies, in areas such as implementation research, which may strengthen public health preparedness. The never-ending debate on the lack of high-quality evidence for facemask use in community settings during COVID-19 is a case in point.

Much of the interventions undertaken by public health practitioners are not evaluated in a scientific manner for want of epidemiological and biostatistical skills [16]; even when evaluated, the research findings are not disseminated and reported in peer reviewed journals as practitioners may not have the time to write manuscripts. These challenges further emphasize the need for medical curricula reform and for a stronger commitment by governments and national institutions to develop and support the field of public health.

## CONCLUSION

There has been a significant increase in life expectancy worldwide. New diagnostic, preventive, and treatment approaches have reduced death rates. However, a high prevalence of chronic disease, emerging and reemerging patterns of infectious disease, and social factors such as health inequity present serious challenges. Additionally, global climate change, natural disasters, rapid urbanization, deforestation and subsequent closer contact with animals, migration, and conflicts are increasing the threat posed by pathogens. A continued focus only on the biomedical, clinical approach to treating disease is a disservice to humanity, especially in light of the staggering – and ever increasing – economic cost of such an approach. Advocating for the role of public health in policy formation and community education; strengthening the public health curriculum in medical education; and increasing the investments in evidence-based public health interventions are actions which are necessary and required. Promoting public health research, particularly in low- and low-middle-income countries to find custom-tailored solutions to local health problems will be invaluable. With COVID-19, the world has paid a tremendous price, both in terms of human suffering and social and economic disruption. Repetition of such an event appears unfortunately not impossible as the world has already witnessed glimpses of such pandemics in the form of severe acute respiratory syndrome (SARS-CoV) and the Middle East respiratory syndrome (MERS-CoV). We can be sure that pandemics will occur again. We cannot face them equally unprepared and equally incapable of a swift and unified global response. The priority placed on public health has been low for far too long. This complacency must end now. It is time to take public health seriously.
